# Low-cost in-house re-formulated brain heart infusion medium for effective planktonic growth and early detection of bloodstream bacterial pathogens

**DOI:** 10.3389/fmicb.2025.1680006

**Published:** 2025-12-02

**Authors:** Jonathan Hira, Nasib Bin Mahbub, Jawad Ali, Rafi Ahmad

**Affiliations:** 1 Department of Biotechnology, University of Inland Norway, Hamar, Norway; 2 Department of Clinical Medicine, Faculty of Health Sciences, UiT, The Arctic University of Norway, Tromsø, Norway

**Keywords:** bacteremia, sepsis - diagnostics, Oxford nanopore sequencing, diagnostics of infectious diseases, metagenomic next- generation sequencing, blood pathogens

## Abstract

Sepsis, a clinically defined life-threatening condition, is a global contributor to high morbidity and mortality rates in humans. It is caused by systemic bloodstream bacterial infections, primarily involving aerobic pathogens such as *Escherichia coli*, *Staphylococcus aureus*, and *Klebsiella pneumoniae*. Rapid and accurate identification of these pathogens is a high-demand task, as prolonged diagnosis may increase the mortality rate among sepsis patients. Globally, commercial blood culture systems like the BD BACTEC™ FX blood culture system, which utilizes BD BACTEC™ PLUS Aerobic/F culture bottles (used in this study), are commonly used to detect aerobic bloodstream infections. However, due to high costs (∼$10.00–$15.00/bottle), limited availability of culture media (especially in low- and middle-income countries, and war zones), and a lack of customization for antibiotic susceptibility assay and epidemiology research, there is a need for secondary alternatives to facilitate the growth and identification of bloodborne pathogens. Therefore, we developed a low-cost (∼$4–$5/bottle) in-house culture medium with a newly improved formulation of Brain Heart Infusion media that enhances bacterial growth from spiked human blood tested on a panel of bacteria (*Escherichia coli*, *Staphylococcus aureus*, *Klebsiella pneumoniae*, *Acinetobacter baumannii*, *Pseudomonas aeruginosa*, and *Enterococcus faecalis*). The growth dynamics of these microbes in in-house formulated BHI-Blood+ culture media coincide with those in BACTEC™ Plus Aerobic/F culture vials, which primarily suggests the compatibility of bloodborne pathogens with this media and can be flagged positive <8 h based on cellular growth rate. Additionally, conventional qPCR-based early detection (<24 h) and validation with the Oxford Nanopore MinION NGS platform highlight the value of this in-house culture media as an alternative to commercial culture media in terms of low-cost availability.

## Introduction

Bloodstream infections (BSIs) are severe conditions caused by the presence of viable pathogens, including bacteria, fungi, or viruses, in the bloodstream, often progressing to sepsis, a life-threatening condition. Sepsis arises when localized infections, such as those in organs, trigger a dysregulated immune response, leading to systemic inflammation, organ dysfunction, and potentially septic shock (Singer et al., 2016). Progression to septic shock involves persistent hypotension and multi-organ failure, with mortality rates above 40% (Evans et al., 2021). Globally, sepsis affects approximately 49 million people annually, causing 11 million deaths, representing 20% of worldwide mortality (Rudd et al., 2020). Low- and middle-income countries (LMICs) bear 85% of sepsis-related deaths due to limited diagnostic and therapeutic resources (Reinhart et al., 2017). In high-income countries, sepsis management incurs significant costs, with the U.S. spending approximately $24 billion annually (Paoli et al., 2018). Timely diagnosis and antimicrobial therapy are critical, as each hour’s delay increases the mortality risk by 7.6% (Kumar et al., 2006). Sepsis is predominantly caused by aerobic bacteria, which significantly contribute to its high morbidity and mortality. *Staphylococcus aureus* accounts for 20%–30% of BSIs, often linked to invasive procedures like catheter use or endocarditis, with methicillin-resistant strains (MRSA) complicating treatment (Tacconelli et al., 2018). *Escherichia coli*, responsible for 25%–30% of BSIs, typically originates from urinary or intra-abdominal infections, with increasing resistance to third-generation cephalosporins (Nordmann et al., 2012). *Klebsiella pneumoniae* (10%–15% of BSIs) is associated with hospital-acquired pneumonia and exhibits growing carbapenem resistance, with mortality rates for resistant strains reaching 40%–50% (Pitout et al., 2019). *Pseudomonas aeruginosa*, *Acinetobacter baumannii*, and *Enterococcus* spp. others are less common (approximately 10% of cases) and pose challenges due to their intrinsic resistance to multiple antibiotics, including carbapenems, making it difficult to treat critically ill patients (Peleg et al., 2008; Cattaneo et al., 2021). These aerobic pathogens thrive in the iron-rich environment of blood, evading host defenses (Cassat and Skaar, 2013). The rise of antimicrobial resistance (AMR) in these organisms exacerbates treatment challenges, necessitating the development of rapid diagnostics and targeted therapies to improve outcomes (Tacconelli et al., 2018).

Traditional blood culture remains the cornerstone for diagnosing BSIs, involving the incubation of patient blood samples in nutrient-rich media to detect microbial growth (Ombelet et al., 2019). Most used automated systems, such as BD BACTEC™, BacT/ALERT, and Thermo Scientific VersaTREK System, enhance detection of positive culture by monitoring CO2 production or pH changes. The average lower limit for time-to-detection (TTD) of positive cultures varies by species and is influenced by the detection system used. For example, in the BD BACTEC™ culture system, the average lower limit for *E. coli* ranges from 9 to 11 h, while in the BacT/ALERT culture system, it is approximately 12.3 h. However, it may take longer depending on the bacterial species (for example, TTD for *Mycobacterium tuberculosis* (MTB) is around 14 days) (Miller et al., 2011; Tyrrell et al., 2012; Li et al., 2019; Ransom et al., 2021; Yarbrough et al., 2021; Yuan et al., 2025). Positive samples undergo subculturing, Gram staining, and further identification by biochemical tests or MALDI-TOF, followed by antimicrobial susceptibility testing (AST), which requires an additional 24–72 h or more (Samuel, 2023). Despite their reliability, these systems face significant challenges. They are time-consuming, often taking 2–5 days for complete pathogen identification and susceptibility profiling, which can delay targeted therapy (Samuel, 2023). High-cost systems (>$10,000), including commercial media (∼$10.00–$15.00/bottle), compounded by supply chain issues and stockouts, limit accessibility, particularly in LMICs (Hardy et al., 2024; Ryder et al., 2024). Contaminated cultures lead to false positives, resulting in increased hospitalization costs of ∼$6,715–$111,627 per patient (Dempsey et al., 2019). Additionally, proprietary formulations lack customization for advanced molecular diagnostics, such as mNGS, hindering protocol-specific adjustments. The limitations of commercial blood culture media, including high costs, limited availability, and a lack of customization, necessitate the development of reformulated, low-cost, and adaptable alternatives. In low- and middle-income countries (LMICs), the high cost of imported bacterial blood culture commercial media restricts access, particularly in rural areas, due to constrained budgets and supply chain challenges (Ombelet et al., 2022; Hardy et al., 2024). Commercial media stockouts, exemplified by the 2024 BD BACTEC™ shortage, hinder timely sepsis diagnosis (Ryder et al., 2024). Additionally, proprietary formulations lack flexibility for protocol-specific adjustments required for advanced diagnostics, such as next-generation sequencing (NGS), as additives like resins alter nutrient dynamics and complicate molecular applications (Ombelet et al., 2019; Buchan, 2022). In-house, customizable media can be tailored to enhance pathogen detection, optimize DNA retrieval, and neutralize inhibitors, improving diagnostic sensitivity (Gu et al., 2019). Affordable formulations promote antimicrobial stewardship, address antimicrobial resistance (AMR), and expand access in resource-limited settings, ultimately enhancing sepsis management and patient outcomes (Hardy et al., 2024). However, the central question arises, “*Which commercial media can be reformulated to be compatible for culturing bloodstream pathogens?*” In this study, BHI medium has been selected for re-formulation purposes. Why, because BHI provides essential nutrients like amino acids, peptides, and carbohydrates, creating an optimal environment for both fastidious and non-fastidious bacteria (Atlas, 2010). Unlike other common bacterial culture media, such as Luria-Bertani (LB), Tryptic Soy Agar (TSA), Columbia Blood Agar, or any other selective media, BHI’s broad-spectrum support makes it ideal for routine blood culture systems, accommodating diverse microbial metabolisms. Compared to BHI’s enriched composition, which better mimics the blood’s nutrient profile, enhancing pathogen recovery in complex samples (Rohner et al., 1991; Atlas, 2010; Bonnet et al., 2019). Its cost-effectiveness is particularly advantageous in LMICs, where commercial media are prohibitively expensive, enabling its demand for culturing bloodstream pathogens (Atlas, 2010). Additionally, BHI’s flexibility allows for customization of advanced diagnostics, such as mNGS, by optimizing nutrient dynamics and neutralizing inhibitors, thereby addressing the limitations of proprietary commercial media (Buchan, 2022). Thus, BHI’s universal applicability and affordability raise the question of its reformulation compatibility with automated systems, such as BD BACTEC™, facilitating rapid growth detection and feasibility for downstream molecular diagnostics, including qPCR and NGS. Therefore, in this study, we aim to investigate whether in-house reformulation of BHI medium can support effective growth of common aerobic pathogens involved in bloodstream infections directly from blood, and secondly, whether the medium is applicable to molecular diagnostics approaches (Ashikawa et al., 2018; Israeli et al., 2020; Avershina et al., 2022; Miyakoshi et al., 2024; Moragues-Solanas et al., 2024; Nielsen et al., 2025).

## Materials and methods

### Ethics statement

In this study, human blood samples were utilized to simulate hospital-grade blood cultures typically obtained from patients. These samples were voluntarily donated by healthy adult individuals who provided informed consent prior to collection at the Department of Biotechnology, University of Inland Norway. The criteria for a healthy blood donor were that each donor had to be healthy, not on any antibiotics, free of recent COVID-19 infection, and have not traveled recently. The study does not intend to use or analyze human DNA; therefore, any sequencing data originating from the human genome were discarded.

### Bacterial strains

In this study, the bacterial strains selected are common aerobic species known to cause bloodstream infections, such as *Escherichia coli* NCTC 13441, *Staphylococcus aureus* CCUG 17621, *Klebsiella pneumoniae* CCUG 225T, *Acinetobacter baumannii* CCUG 19096T, *Pseudomonas aeruginosa* CCUG 17619, and *Enterococcus faecalis* CCUG 9997. All bacterial strains were revived from glycerol stock and cultivated in BHI agar medium at 37 °C for 24 h prior to isolation of single colony isolates.

### BHI-Blood+: reformulation of BHI and broth preparation

To enhance the growth of bacteria in blood, Brain Heart Infusion Broth (BHI) media (53286, Sigma-Aldrich) was supplemented with yeast extract (Y1625, Sigma-Aldrich), L- (−)-Norepinephrine (+)-bitartrate salt monohydrate (norepinephrine) (A9512, Sigma-Aldrich) vitamin mix (MBD0063, Sigma-Aldrich) and polyanetholesulfonic acid sodium salt (SPS) (P2008, Sigma-Aldrich). In-house reformulated BHI was prepared in a two-step process: (1) BHI powder was dissolved in distilled water according to the manufacturer’s instructions. Prior to autoclaving, yeast extract was added at a final concentration of 0.5%. After the autoclave, the media were acclimatized to room temperature. Norepinephrine and SPS stock were prepared according to the manufacturer’s instructions. Norepinephrine and SPS were sterile filtered through a 0.1 μm syringe filter to prevent mycoplasma contamination. Finally, norepinephrine (final concentration, 300 μM), vitamin mix (final concentration 2%, comprises Biotin: 2 mg/L, Folic acid: 2 mg/L, Pyridoxamine-HCl: 10 mg/L, Thiamine-HCl × 2 H_2_O: 5 mg/L, Riboflavin: 5 mg/L, Nicotinic acid: 5 mg/L, D-Ca-pantothenate: 5 mg/L, Cyanocobalamine: 0.1 mg/L, p-Aminobenzoic acid: 5 mg/L, Lipoic acid: 5 mg/L, KH_2_PO_4_: 900 mg/L), and SPS (final concentration, 0.05%) were added to the BHI-Yeast broth. For the scope of this literature, this reformulated, in-house developed BHI media is termed BHI-Blood+.

### Bacterial planktonic growth kinetics in BHI-blood+

Bacterial growth kinetics were measured in BHI-Blood+ compared to standard BHI medium to evaluate the planktonic growth performance of various bacterial strains. Bacterial growth kinetics parameters, including lag time and growth rate, were measured as corresponding parameters using the standard microdilution method. Before growth kinetics measurements, bacterial strains were prepared accordingly to the following steps. Bacterial strains were revived from glycerol stock and isolated as single colonies from an overnight static growth culture on BHI agar. Individual colony from each strain was inoculated in 3 mL of BHI broth and cultivated for 2–3 h with moderate shaking at 37 °C, depending on the strain’s background. After brief cultivation, the culture samples were centrifuged at 5000 rpm for 10 min. The supernatant was discarded, and the pellets were resuspended in PBS. Bacterial cell density was adjusted to ∼10^5^ CFU/ml and seeded into each well of a 96-well plate at the final concentration. The Breath-Easy sealing membrane (Z380059, Merck) was used to seal the plates, which were then incubated at 37 °C in a Synergy H1 microplate reader. Absorbance at 600 nm for each well was measured at an interval of 30 min over 24 h. Acquired growth data were analyzed using QurvE software v1.1 (Wirth et al., 2023). Raw data generated from the microplate reader were transformed to fit into QurvE, and parameters such as growth OD threshold, time at 0 h (t_0_), and maximum time for growth (t_max_) were set during a quality check on the raw growth curve. The growth threshold was set to 1.5, and t_max_ to 24 h to extract growth kinetics parameters. Growth profiling of individual bacteria was performed using a growth-fitting exponential growth model with a heuristic linear regression method on log-transformed data (Hall et al., 2014). This model determines maximum growth rates (μ_max_) for individual bacteria cultivated in growth medium. A linear fit was performed using an R2 threshold of 0.95, a Relative standard deviation (RSD) of 0.1, and a dY threshold of 0.05 to estimate the difference in maximum growth and minimum growth. Maximum growth rate (μ_max_) and lag time (λ) were extracted from the linear growth profiling model. μ_max_ was validated using a Gompertz parametric fit (Kahm et al., 2010), and lag time λ was validated using a spline fit model (Kahm et al., 2010).

### Profiling bacterial static growth in BHI-blood+

#### BHI-blood+ spiking

Blood samples were collected from healthy volunteers via the sterile venipuncture method. Samples were immediately prepared for culturing to avoid any contamination. Two parallel experimental conditions were established to evaluate bacterial growth dynamics: one using the in-house formulated blood culture medium BHI-Blood+ and the other employing commercially sourced BD BACTEC™ Plus Aerobic/F culture vials (Becton Dickinson and Company, Franklin Lakes, NJ, USA). Before spiking blood, all reference strains were revived from glycerol stock, and an individual isolated colony was inoculated in 3 mL of BHI. Inoculated strains were incubated at 37 °C for 2–3 h with mild agitation to achieve the early exponential growth phase. After short cultivation, culture samples were centrifuged at 5000 rpm for 10 min. Supernatant was discarded to remove standard BHI medium contaminants and any extracellular growth factors. Pellets were washed and resuspended in PBS. The optical density (OD) of each bacterial culture was quantified at 600 nm using a UV-3100PC Spectrophotometer (VWR Life Science, USA), and the CFU/ml was adjusted to a concentration that resulted in a final concentration of ∼10^2^ CFU/ml when spiked into the blood. The spiked bacterial concentration was verified with the serial dilution colony-forming unit (CFU) method. 5 mL of donated human blood, spiked with bacteria, was added to an equal volume of the formulated medium and BD BACTEC™ PLUS Aerobic/F culture bottles under sterile conditions. Culture bottles were incubated at 37 °C with mild agitation to ensure sufficient oxygen exposure and homogeneity.

#### Droplet CFU quantification

Time-Resolved Droplet-based CFU Quantification method was introduced to profile the static growth performance of all 6 strains and compare with a commercial standard blood culture medium, BD BACTEC™ PLUS Aerobic/F culture bottles. The incubation time varied based on bacterial type: for *E. coli*, *K. pneumoniae*, *A. baumannii*, and *E. faecalis*, samples were collected at 2 and 4 h, whereas *P. aeruginosa* and *S. aureus* required sampling at 2, 4, and 6 h due to their comparatively slow growth phenotypes within these experimental parameters. The early time points were chosen to assess growth performance between the commercial BACTEC™ PLUS Aerobic/F culture and BHI-Blood+ and the ability to track early cell proliferation. At each targeted time point, 2 mL samples were aseptically taken from each culture bottle. These samples underwent serial dilution in sterile PBS. 10 μl droplets with replicates from each dilution were plated on standard BHI-agar plates and incubated overnight at 37 °C. The colonies were later counted to calculate the CFU/mL at each time point, allowing for a quantitative evaluation of the time-resolved bacterial growth progression. Both short-incubation and cultured blood samples were immediately stored at −20 °C for use in the downstream experimental method.

### Molecular detection of pathogen in BHI-blood+

#### DNA extraction

Blood samples spiked with bacterial strains were collected from BHI-Blood+ at specific incubation points: (a) 4 h for *E. coli*, *K. pneumoniae*, *A. baumannii*, and *E. faecalis*, and (b) 6 h for *P. aeruginosa* and *S. aureus*. Additionally, pure isolates and 24-h cultures were also subjected to DNA extraction, and these samples were used as qualitative controls for the early detection strategy. From each of the 2 ml collected samples, DNA was extracted using the QIAamp Biostic Bacteremia DNA Kit (QIAGEN, Hilden, Germany) according to the manufacturer’s instructions. DNA was eluted in nuclease-free water and quantified with the Qubit dsDNA HS Assay Kit on a Qubit 4 Fluorometer (Thermo Fisher). The purity of DNA samples was checked using the NanoDrop ND-1000 Spectrophotometer (Thermo Fisher) to measure the OD 260/280 ratio of 1.8 and the OD 260/230 ratio of 2.0–2.2. If necessary, optional concentration and purification were performed with the Agencourt AMPure XP system (Beckman Coulter, USA). Extracted DNA samples were subjected to qPCR-based pathogen detection, followed by Oxford nanopore based sequencing.

#### Real-time quantitative polymerase chain reaction

To detect bacteria in the extracted DNA samples, Real-time quantitative polymerase chain reaction (qPCR) was utilized. In this study, species-specific primers were used to detect species that were spiked in blood. Detailed primer sequences and references are listed in the Supplementary Table 1. qPCR were performed on a 7500 Fast Real-Time PCR System (Applied Biosystems, Thermo Fisher Scientific, Waltham, MA, USA), employing the following thermal profile: an initial denaturation stage at 95 °C for 12 min to activate the Hot FIREPol enzyme, followed by 40 cycles comprising denaturation at 95 °C for 25 s, annealing at 60 °C for 45 s, and an extension phase at 72 °C for 1 min. Cycle threshold (Ct) values were recorded for each reaction and used for qualitative evaluation of the presence of bacterial DNA in extracted samples. Mean Ct values were calculated from replicates and visualized differences using an in-house developed R Shiny app. The interpretation of early detection was performed based on ΔCT differences between 4 and 6 h culture samples, pure isolated, and 24 h culture samples.

#### Oxford nanopore sequencing

In this study, Oxford nanopore sequencing was conducted on 4 or 6-h culture samples to evaluate the possibility of early detection of spiked species and whether BHI-Blood+ can be used as a routine blood culture medium for detecting bacteria using a next-generation sequencing platform. AMPure XP-purified DNA samples were subjected to library preparation using the Rapid Barcoding Kit 96 V14 (SQK-RBK114.96, Oxford Nanopore Technologies, Oxford, UK), which facilitated the multiplexing of samples by incorporating distinct barcodes into each DNA sample. The library preparation was carried out according to the manufacturer’s protocol without any modifications. The prepared sample library was loaded onto the MinION Flow Cell (R10.4.1, FLO-MIN114, Oxford Nanopore Technologies, Oxford, UK) and sequenced using the MinION MK1D device. The parameters for the sequencing run were set to Fast model v4.3.0 basecalling, implemented by Dorado 7.6.8, with a Minimum Q score of 7 and a run time of 24 h. The output files generated by the MinKNOW software were analyzed using minimap2 v2.28-r1209 based alignment to the reference genome. The number of reads and genome coverage of spiked strains were estimated to confirm the detection and identity of the spiked bacterial species. The reference assemblies for the strains used in this study can be found in the NCBI database by using the accession numbers *E. coli* NCTC 13441 (GCA_900448475.1), *P. aeruginosa* CCUG 17619 (GCA_024507955.1), *K. pneumoniae* CCUG 225T (GCA_000742135.1), *A. baumannii* CCUG 19096T (GCA_900444725.1), *S. aureus* CCUG 17621 (GCA_028596245.1), and *E. faecalis* CCUG 9997 (GCA_022406475.1). Reads aligned to reference bacterial genomes were subjected to iterative BLASTN searches to confirm the species and identify false positives.

### Statistical analysis

Statistical analysis on both growth profile parameters was performed using GraphPad Prism (version 10.5.0). A non-parametric Mann-Whitney U test was applied to both planktonic and static bacterial growth profiles. A two-tailed *p*-value of <0.01 was considered statistically significant.

## Results

### Impact of BHI-blood+ cocktail on bacterial planktonic growth: no detrimental effect observed

**FIGURE 1:**
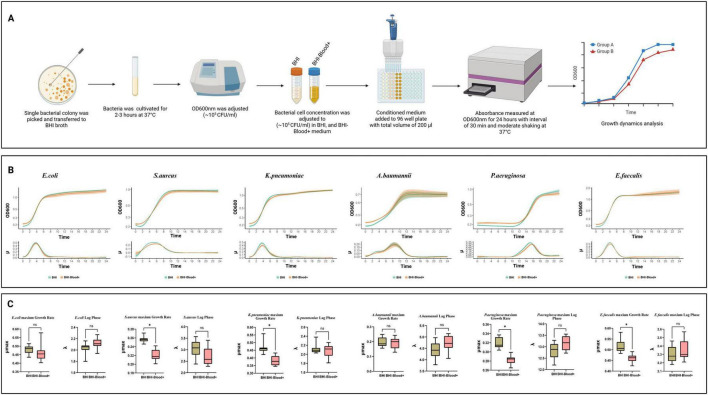
An in-depth analysis of bacterial growth dynamics of six species, including *Escherichia coli*, *Staphylococcus aureus*, *Klebsiella pneumoniae*, *Acinetobacter baumannii*, *Pseudomonas aeruginosa*, and *Enterococcus faecalis*, under two culture conditions: **(A)** Overview of experimental workflow for bacterial planktonic growth dynamics in BHI and BHI-Blood+ supplemented with norepinephrine, vitamin mix, and SPS. **(B)** Each panel includes a growth curve representing planktonic growth over 24 h, **(C)** a growth rate distribution, and a lag phase, as analyzed by QurvE on log-transformed data. Box plots indicate statistical comparisons with no significant (ns) labels, where *p* ≤ 0.01 and significant differences are marked with asterisks (*).

Figure 1A illustrates an overview of the experimental procedure to perform the planktonic growth profile of all 6 strains in BHI-Blood+. Profiling *E. coli* planktonic growth under various conditions provides a deeper understanding of the effects of norepinephrine, a vitamin mix, and an SPS cocktail. Growth curves, depicted as log growth (OD600) against time, reveal distinct phases: an initial lag phase during which bacterial adaptation occurs, followed by an exponential growth phase that peaks approximately 2–8 h later, and a subsequent stationary phase beginning at 9 h and continuing thereafter Figure 1B. When the two conditions, BHI and BHI-Blood+ are compared, the curves of both conditions show overlapping trajectories, indicating no significant deviation in growth patterns. Quantitative analysis of the lag phase and the maximum growth rate (μ_max_), measured with statistical significance (*p* < 0.01) between BHI and BHI-Blood+ conditions, shows no substantial differences between both conditions and therefore supports the absence of inhibitory effects exerted by BHI-Blood+ additives Figure 1C.

*Staphylococcus aureus*, on the other hand, exhibits a distinct growth dynamic compared to *E. coli* when reacting to the additives of BHI-Blood+ Figure 1C. While the lag phase shows no significant differences between BHI and BHI-Blood+, the maximum growth rate (μ_max_) of *S. aureus* under BHI conditions dominates that of BHI-Blood+. Growth rate results also support these findings, indicating that a lower shift in μ_max_ occurred after 5 h. While the growth rate of BHI peaked at late 4 h, both conditions overlapped in the exponential phase between 2 and 8 h, Figure 1B. These findings suggest that while the additives do not alter the lag phase, the reduction in μ_max_ in BHI-Blood+ may be due to a metabolic adaptation in the presence of these additives.

A similar trend of growth trajectories was also observed for *K. pneumoniae*, Figure 1B. Similar adaptation periods were observed across both conditions, supporting non-significant differences in the lag phase, as shown in Figure 1C. However, the μ_max_ rates showed a significant reduction in BHI-Blood+ compared to BHI, suggesting that the peak growth capacity of *K. pneumoniae* is impaired by the additives, Figure 1C. Additionally, the growth curves for *K. pneumoniae* display an exponential growth phase that overlapped in both conditions, supporting a minimal effect of additives on bacterial growth kinetics Figure 1B. *E. faecalis* demonstrates similar trends to *S. aureus* and *K. pneumoniae* in showing differences in μ_max_, Figure 1C. However, there were no significant differences in the lag phase. In contrast, the growth behavior of *A. baumannii* exhibited no significant difference in either the lag phase or growth rate, implying that the additives do not alter its peak planktonic growth capacity, Figure 1C. These findings are also supported by a biphasic exponential growth pattern, overlapping between both conditions in *A. baumannii* (exponential phases, 2–4 and 4–6 h), which depicts consistent metabolic adaptation across conditions, Figure 1B. On the other hand, compared to all other strains, *P. aeruginosa* demonstrates a prolonged lag time when cultivated with both media, Figure 1B, while significantly differing from each other in maximum growth rate, Figure 1C.

### Compatibility between the BHI-blood+ medium and the BD BACTEC™ for short cultivation

**FIGURE 2:**
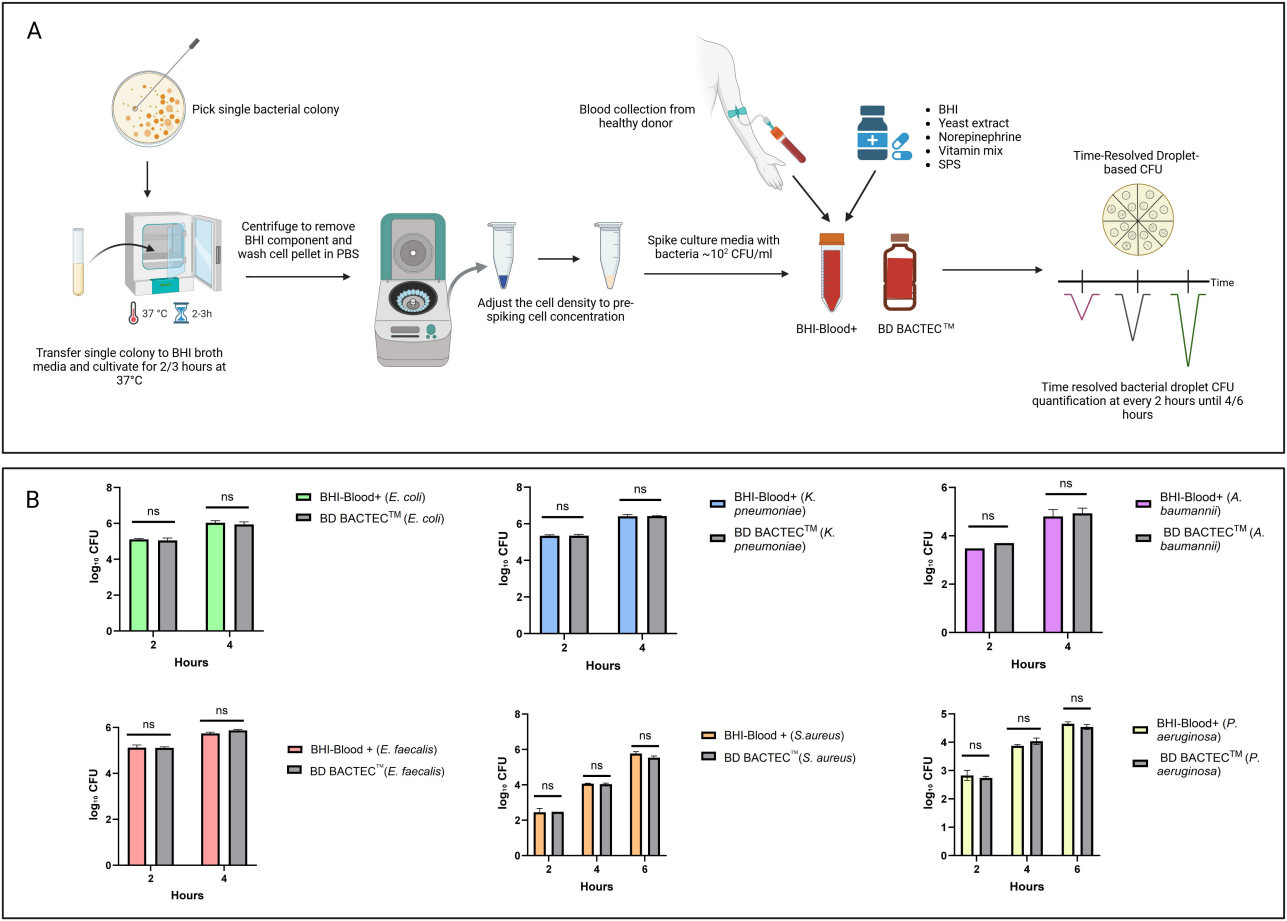
**(A)** Illustrates experimental overview of profiling bacterial static growth dynamics. **(B)** The growth dynamics of 4 bacterial species – *E. coli*, *K. pneumoniae*, *E. faecalis*, *A. baumannii*– were evaluated using BHI-Blood+ and BD BACTEC™ PLUS Aerobic/F culture media over a period of 2 and 4 h, whereas *P. aeruginosa* and *S. aureus* for 2, 4, and 6 h, with log_10_ CFU/ml counts measured at intervals of 2 h. Statistical analysis showed no significant difference (ns) between the two conditions at each time.

Figure 2A depicts bacterial time-resolved droplet-based CFU quantification assessment of static growth profile of six clinically significant pathogens: *E. coli*, *K. pneumoniae*, *P. aeruginosa*, *E. faecalis*, *A. baumannii*, and *S. aureus*, in two culture media: BHI-Blood+ and BACTEC™ PLUS Aerobic/F culture. Raw CFU counts compared between BHI-Blood+ and BD BACTEC™ at different time points for all the strains are presented in Supplementary Table 2, and agar plate images captured are shown in Supplementary Figure 1. CFU quantification results suggest that *E. coli*, with average values of ∼10^5^ CFU/mL at 2 h and ∼10^6^ CFU/mL at 4 h, highlights the in-house medium’s equivalent performance to BD BACTEC™, without any significant difference between the two media Figure 2B. Similarly, *K. pneumoniae* and *E. faecalis* grew rapidly over a 4-h period Figure 2B. Starting at ∼10^2^ CFU/mL, the BHI-Blood+ supported growth to ∼10^5^ CFU/mL by 2 h and ∼10^6^ CFU/mL by 4 h, while BACTEC™ PLUS Aerobic/F culture media achieved similar CFU/mL compared to BHI-Blood+ without statistical significance, confirming the efficacy of BHI-Blood+ for the early detection of these pathogens. However, *A. baumannii* demonstrated ∼10ł CFU/mL at 2 h and increased to ∼105 CFU/mL by 4 h Figure 2B, which was slower static growth as compared to *E. coli*, *K. pneumoniae*, and *E. faecalis*, but when compared to BACTEC™ PLUS Aerobic/F culture media, BHI-Blood+ also showed its compatibility for *A. baumannii* growth in blood and no significant differences.

On the other hand, the delayed static growth dynamics of both *P. aeruginosa* and *S. aureus* were assessed at an additional time point, 6 h Figure 2B. *P. aeruginosa* growth progressed over 6 h, starting at ∼10ł CFU/mL at 2 h, slowly rising to 104 CFU/mL by 4 h, and reaching ∼105 CFU/mL by 6 h in both media. Contrary to this, *S. aureus* showed limited proliferation at 2 h (∼10^2^ CFU/mL) in both media, increasing to the ∼104 CFU/mL range by 4 h. By 6 h, the BHI-Blood+ supported a higher bacterial load (∼106 CFU/mL), with no statistical differences compared to BACTEC™ PLUS Aerobic/F culture media Figure 2B.

These results demonstrate that BHI-Blood+ and BD BACTEC™ PLUS Aerobic/F culture media support comparable early-phase bacterial proliferation *in vitro* across a wide range of Gram-negative and Gram-positive organisms, indicating that BHI-Blood+ is compatible with bloodborne pathogens.

### BHI-blood+ supports bacterial early detection

**FIGURE 3:**
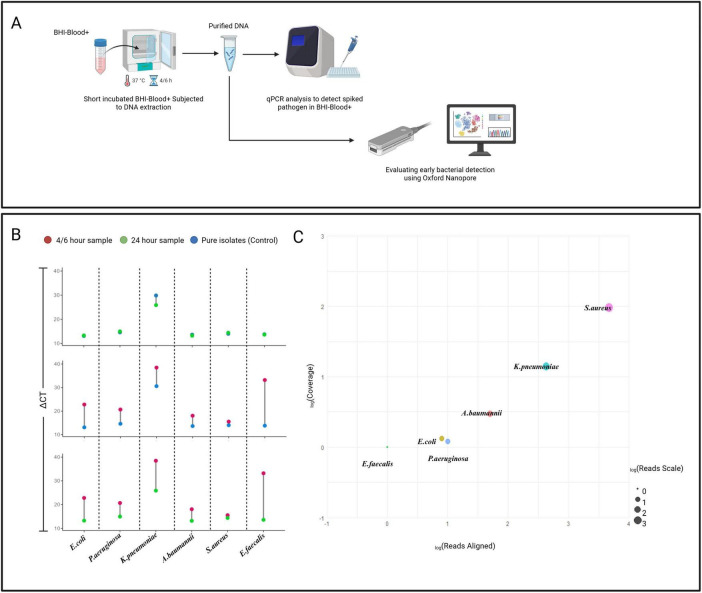
**(A)** Illustrates experimental workflow for molecular detection of targeted species in BHI-Blood+ medium. **(B)** Depicts the analysis of species-specific detection across different samples spiked by bacterial strains. ΔCt differences between control and culture conditions demonstrate earlier detection efficiency. Samples collected at 4 h (Red dots), 24 h (Green dots), and pure isolates (Blue dots). **(C)** Displays a bubble plot illustrating the relationship between log-transformed sequencing reads aligned to reference genome (*X* axis = _*log*_Reads) and Coverage (*Y* axis = _*log*_Coverage). The bubble size (based _*log*_Reads scale) represents the correlation between read count and coverage, with larger bubbles indicating a higher ratio of read count to coverage, signifying enhanced species detection.

Figure 3A demonstrates molecular detection strategy for pathogen spiked in both BHI-Blood+ and BD BACTEC™ PLUS Aerobic/F culture media. DNA extracted from blood cultures spiked with six sepsis-relevant bacterial strains (*E. coli*, *P. aeruginosa*, *K. pneumoniae*, *A. baumannii*, *S. aureus*, and *E. faecalis*) using the BiOstic protocol showed high purity via NanoDrop measurements, confirming an efficient extraction process. To assess whether BHI-Blood+ supports early detection through short incubation periods, each strain was analyzed at specific time points: *E. coli* (4 and 24 h), *P. aeruginosa* (6 and 24 h), *K. pneumoniae* (4 and 24 h), *A. baumannii* (4 and 24 h), *S. aureus* (6 and 24 h), and *E. faecalis* (4 and 24 h). Mean Ct value differences were evaluated to infer detection of bacterial proliferation in spiked samples.

Results, summarized as Ct values in Supplementary Tables 3–6, indicate that all six strains were detected at their respective time points after short incubation. Figure 3BΔCt values between short incubation and positive controls showed early detection for all strains except *E. faecalis*, which exhibited higher ΔCt values. The detection template generated from pure isolates, and 24-h sample defines the early detection efficiency. As 24-h samples provided sufficient biomass for the detection of all species, reflected by the lowest ΔCt values when compared to pure isolates. Comparison of short incubation and 24-h cultures revealed trajectories sufficient to confirm cellular proliferation of these strains earlier than 24 h.

On the other hand, minimap2 analysis reports the number of reads (Accession: PRJNA1295148) aligned to the reference genome derived from nanopore sequencing and significant *E*-value to the designated species validates the species detection Figure 3C (summarized in Supplementary Table 7). Sequencing results confirm the detection of all targeted species except *E. faecalis*, and the reason for this detection failure is still unclear. However, results from both planktonic and static growth profiles suggest that errors may arise during DNA extraction or sample library preparation, which would be difficult to trace because the samples were not subjected to host depletion. Among the species, *S. aureus* and *K. pneumoniae* depict the highest read counts and coverage. In contrast, lower read counts for *E. coli*, *P. aeruginosa*, and *A. baumannii* indicate potential technical issues, such as low quality of sequencing due to short-reads generated by the DNA extraction process itself, higher level of host DNA contamination, and importantly, the DNA extraction chemistry may not be optimal for short incubation sample condition, which represents high host DNA compared to low bacterial DNA concentration. Overall, both qPCR and nanopore sequencing validate the compatibility of BHI-Blood+ media for early bacterial detection.

## Discussion

### BHI-Blood+: a cost-effective alternative for blood culture diagnostics in resource-limited settings

Blood is generally considered a sterile environment, free from viable pathogens under healthy conditions. However, commensal microbes can be translocated transiently and sporadically from different body sites into the bloodstream (Tan et al., 2023). Even a few percent of pathogens that reach the bloodstream, opsonization and phagocytosis-based immune responses actively suppress the growth of these pathogens. These host defense mechanism releases multiple inhibitors to create a hostile environment for pathogens (Uribe-Querol and Rosales, 2020). Commercial blood culture media are formulated in such a way that they not only neutralize the inhibitors released by host immune responses but also provide nutrients and growth factors to support the recovery of pathogens, facilitating accurate sepsis diagnosis. This culture system is regarded as the gold standard for detecting bloodstream infections. BD BACTEC™ comprises ingredients, which include Soybean-Casein digest broth, yeast extract, amino acids, sugar, vitamins, SPS, antioxidants/reductants, nonionic adsorbing resin, and cationic exchange resin, which effectively support the growth of sepsis-inducing pathogens. While these systems support the growth of pathogens, they are yet limited by high costs and supply shortages (Itoh et al., 2025). For instance, the cost of BD BACTEC™ Plus Aerobic/F bottles typically ranges from ∼$10–$15 per bottle (30 ml), depending on the suppliers and import expenses in low-resource environments (Fisher Scientific, 2025). Addressing the limitations of commercial blood culture media, the need for an alternative has led to the reformulation of the existing BHI media. This study evidently demonstrated that use of blood-supported, in-house formulated brain heart infusion (BHI-Blood+) offers similar growth dynamics efficacy of sepsis inducing aerobic pathogens, thus receiving attention as a potential alternative to commercial media. One significant advantage of BHI-Blood+ is that, in addition to utilizing locally sourced BHI, yeast extract, and other additives, it is estimated to cost ∼$4–$5 per bottle (30 ml). Such a significant cost reduction enhances accessibility in resource-constrained settings without compromising bacterial growth performance. Additionally, the additives in BHI-Blood+ comprise 4–5 ingredients (yeast extract, norepinephrine, vitamin mix, and SPS), compared to 8–9 ingredients in BD BACTEC™ Plus Aerobic/F bottles, offering an attractive option for hospitals and clinics in developing countries, where budget constraints often limit access to advanced diagnostic tools.

On the other hand, the individual supplements added in BHI-Blood+ are classical requirements for bacterial growth. Starting with yeast extract, which provides essential amino acids, peptides, and vitamins, supporting the metabolic needs of individual bacteria (Tao et al., 2023). Vitamin mix used in BHI-Blood+ is to supplement additional cofactors to promote bacterial growth in BHI-Blood+ (Agostino et al., 2020; Pham et al., 2021; de Raad et al., 2022). However, one of the most essential add-ons in BHI-Blood+ is norepinephrine, a group of hormones and neurotransmitters known as Catecholamines. Catecholamines are released into the bloodstream in response to stress, regulating multiple physiological functions, particularly by escalating blood pressure and increasing cardiac output (Eisenhofer et al., 2004; Belfiore et al., 2025). Notably, some studies have depicted the release of catecholamines in the bloodstream as the body’s acute response to infection and play an important role in maintaining vascular tone, heart rate, and blood pressure to counteract the hypotension and tissue hypoperfusion characteristics during septic shock (Belfiore et al., 2025). However, some studies have also shown that catecholamines, especially norepinephrine, are found to promote bacterial growth by facilitating the acquisition of iron from the host through interaction with host proteins such as transferrin. For example, it has been reported that norepinephrine promotes the proliferation and virulence of pathogenic bacteria in the iron-limited environment (Kinney et al., 2000; Sandrini et al., 2014; Niu et al., 2023; Beata et al., 2021). Besides, blood can also be considered an iron-limiting environment, particularly during infection or inflammation. Hepcidin, a hormone, plays a critical role in reducing the amount of iron entering the bloodstream during infection and inflammation, which leads to starvation of pathogens of iron in the bloodstream (Cassat and Skaar, 2013; Michels et al., 2017; Tan et al., 2021). Therefore, during bacterial culture in blood, catecholamines, especially norepinephrine, were supplemented in BHI-Blood+ to accelerate bacterial growth. Moreover, SPS is a cornerstone component in BHI-Blood+, enhancing pathogen recovery by neutralizing host inhibitors and preventing clotting (Palarasah et al., 2010). Thus, the synergistic effect of all these components in BHI-Blood+ creates an optimal growth environment for bloodborne bacteria. This makes it a viable option for clinical microbiological laboratories, especially in resource-limited environments. Due to its open formulation, it has the potential to further customize BHI-Blood+, thereby enhancing its demand. Unlike standardized and exclusive commercial media, BHI-Blood+ can be adapted to specific local needs. For example, laboratories can adjust supplements to optimize the growth of specific pathogens present in their region (Zha et al., 2025). Additionally, BHI-Blood+ offers an advantage in terms of ease of preparation, accessibility, and feasibility in the supply chain. BHI-Blood+ can be easily prepared in laboratories using standard equipment and locally available materials, such as individual components of BHI or commercially available, pre-prepared BHI powders, and additives like vitamin mix, norepinephrine, and SPS. This local production minimizes dependence on international suppliers, which often face delays or disturbances in low-resource environments due to logistical challenges or import restrictions. With such consistent local production, it ensures a steady supply and flexible volume preparation tailored to the needs of internal diagnostics or research, thereby supporting uninterrupted diagnostic services. BHI-Blood+ media is designed to be prepared and used with standard laboratory equipment, such as autoclaves, incubators, and basic microbiological tools, which are typically available in most clinical laboratories, even in low-resource environments. Moreover, unlike BD BACTEC™ or any commercial automated systems, which demand specialized equipment and skilled staff, preparation of BHI-Blood+ does not require significant investments in technology or training for its core functionality–culturing and enhancing pathogens for downstream identification. However, it is important to note that pathogens cultivated in clinical settings require identification using molecular techniques, the most common of which are qPCR or MALDI-TOF. While qPCR machines or standard thermocyclers are commonly used in clinical labs for disease diagnosis, MALDI-TOF systems are quite expensive and not easily accessible. To address the question of whether the pathogens cultivated in BHI-Blood+ can be detected using standard qPCR, we affirm the compatibility of BHI-Blood+ in this regard. Additionally, we mention the compatibility of BHI-Blood+ for pathogen surveillance using next-generation sequencing. Thus, we believe that in any moderate or even low-resource setting where qPCR is not available, BHI-Blood+ can still effectively support the growth of pathogens without any adverse impact. Its adaptability enables labs to scale production to meet diverse demands, independent of pre-packaged commercial products (Orekan et al., 2021). In contrast to commercial systems like BD BACTEC™, a notable limitation of BHI-Blood+ is its lack of adaptability to any automated culture system equipped with automatic detection for positive and negative cultures. Interestingly, this limitation presents a new avenue for research on how BHI-Blood+ can be integrated into existing automated blood culture systems to serve as a substitute for BD BACTEC™ culture bottles when their supply is limited.

### BHI-blood+ is compatible with rapid sepsis diagnostics and reproducible host-pathogen research

Traditional blood culture strategies require 24–72 h for detectable bacterial growth, often making them too slow for critical cases. Thus, delayed treatment in sepsis can cause an increase in mortality risk of 7%–8% per hour. A study done by Ali et al. (2024) demonstrated that shorter blood culture can be achieved with commercial BD BACTEC™ and thereby accelerating blood sepsis diagnosis from sample collection to identify pathogens and ARGs within a 7–9-h timeframe. This study has opened a novel pathogen detection strategy, by using short cultivation to grow bacteria at the exponential phase and using molecular approaches such as mNGS to enable the early detection of pathogens. According to Ali et al. (2024), the BD BACTEC™ system supports a short cultivation strategy. Additionally, BHI-Blood+ supplemented with norepinephrine, vitamin mix, and SPS sheds light on it as a promising medium for short-term incubation, as demonstrated in this study. All pathogens used in this study have been shown to adapt to the short-term cultivation strategy. This study has shed light on the possibility of earlier detection of bacteria through a simple cultivation strategy. In principle, BACTEC™ Plus Aerobic/F culture vials work as microorganisms in the medium proliferate, CO_2_ levels rise as a result of the metabolism of substrates present in the medium. The vial’s integrated sensors detect increased CO_2_ levels through enhanced fluorescence, which is apparently monitored by the BD BACTEC™ FX system (Rohner et al., 1996). The BACTEC™ FX system can flag positive culture for most bloodstream infection pathogens when the CFU reaches >10^7^–10^8^ CFU/ml (Wang et al., 2015), which indicates that detecting certain levels of CO_2_ can take more than 12 h or even longer, even if bacterial growth is in the exponential phase. This study has reproduced the findings of Ali et al. (2024) that the early detection of pathogens can be done by tracking their early proliferation in culture medium. Compared to BD BACTEC™, BHI-Blood+ demonstrated its compatibility for early detection at the cellular level at <8 h. Additionally, the medium’s compatibility with molecular platforms like qPCR and nanopore sequencing positions it as a valuable tool for molecular-based pathogen detection at <24 h, and metagenomic studies for pathogen surveillance. Thus, as a low-cost alternative in-house media, BHI-Blood+ demonstrated its potential as a component in rapid sepsis diagnostics research. However, to demonstrate its full potential, challenges remain in evaluating anaerobes fastidious organisms, or fungi, which may require specialized media or conditions (such as anaerobic atmospheres or additional growth factors) that are not provided by the current formulation. We acknowledge this limitation of BHI-Blood+, as its current composition and preparation are tailored for aerobic pathogens, and caution should be exercised when considering its use for anaerobic pathogens. Moreover, a small subset of bacteria implicated in bloodstream infections may be difficult or impossible to culture using standard or even specialized media, including BD BACTEC™. In such cases, molecular techniques like qPCR or metagenomic sequencing may be necessary for detection. We also evaluated BHI-Blood+ for its compatibility with next-generation sequencing, which suggests using BHI-Blood+ for rare pathogen surveillance. Additionally, clinical validation of real patient samples with potential inhibitors (antimicrobials), cost barriers in resource-limited settings, and feasibility in hospital automation and upscaling adds more questions to address for future studies. In summary, while BHI-Blood+ can be a viable option in the event of supply issues with commercial blood culture media, it should not be regarded as a complete replacement. On the other hand, apart from sepsis diagnosis, BHI-Blood+ can also offer another advantage in addressing the necessities of optimizing culture media for reproducible antibiotic sensitivity tests directly in blood cultures (Bebell and Muiru, 2014; MacVane and Dwivedi, 2024). Previous studies have demonstrated that blood culture resuscitation in culture media can support direct blood culture susceptibility studies, yielding results comparable to those obtained with standard methods, thereby reducing the time to actionable results (Wu et al., 2019). Adding BHI-Blood+ to this extent of capability can be valuable in situations where rapid sensitivity tests are important for patient management. Additionally, antibiotic sensitivity tests using BHI-Blood+ have the potential to support reproducible research in understanding complex host-pathogen interactions in bloodstream infections and their molecular mechanisms of antibiotic resistance. As discussed earlier, BD BACTEC™ is only used for commercial sepsis detection systems, and its use in research is not straightforward, as it is limited to customization or open formulation. Moreover, to the best of our knowledge, commercial human blood culture media and systems are commonly used by the veterinary and agricultural communities (Ogrodny et al., 2023; Pas et al., 2024). Thus, BHI-Blood+ can offer low-cost alternatives, and its open formulation can aid the community in investigating animal-specific bacteremia and the discovery of novel sepsis-causing pathogens. Overall, this study has opened new opportunities for researchers to utilize BHI-Blood+ medium as a routine, low-cost culture system to study the molecular mechanisms of bloodstream pathogens and their interactions with the host. This open formulation can give researchers the advantage of further formulating the medium and upgrading it according to their specific research needs, offering greater flexibility in reproducible research compared to the more restrictive BD BACTEC™ system.

### Limitations

This study highlights BHI-Blood+ as a promising, low-cost alternative to commercial blood culture media, particularly in situations where availability is limited. While BHI-Blood+ demonstrates a satisfactory growth profile for common bloodstream pathogens, it has key limitations that warrant caution and suggest areas for future research. The first limitation is that the evaluation was restricted to aerobic bacteria, leaving the performance for anaerobes, fastidious organisms, and fungi–relevant components of polymicrobial bloodstream infections (BSIs)–untested. To address this limitation, BHI-Blood+ would require additional formulation, as even commercial blood culture bottles often include options for anaerobic and fungal growth. A second limitation is that the selection of clinically relevant pathogens for this study was based solely on their known growth profiles. While understanding the growth characteristics of known reference strains is important for comparisons across media, the potential of BHI-Blood+ could be further enhanced by evaluating clinical strains collected from sepsis cases, including those treated with antibiotics. We acknowledge these limitations and suggest that future studies should explore the adaptations needed for anaerobes and fungi, investigate polymicrobial scenarios, examine antibiotic-exposed clinical samples, and assess automation compatibility to improve sepsis diagnostics globally.

## Conclusion

In conclusion, BHI-Blood+ demonstrates an alternative to commercial blood culture media, especially when supplies of commercial blood culture bottles are limited. It is not intended to replace routine methods. Importantly, BHI-Blood+ addresses key barriers to blood culture diagnostics, such as cost, accessibility, and customization. It has demonstrated compatibility for the rapid detection of sepsis. Its potential to enhance sepsis diagnostics and improve reproducible research practices in resource-limited settings highlights the necessity for further validation and optimization to fully realize its capabilities across various clinical and research applications.

## Data Availability

The sequenced dataset used in this study is available in the online NCBI repository under accession number PRJNA1295148.
