# Supplementary material for: Low-cost in-house re-formulated brain heart infusion medium for effective planktonic growth and early detection of bloodstream bacterial pathogens
Source: Front Microbiol. 2025 Dec 2;16:1680006. doi: 10.3389/fmicb.2025.1680006 (PMC12705537; doi:10.3389/fmicb.2025.1680006)
Supplement: Supplementary file 1 [file Data_Sheet_1.pdf]

Supplementary

Low Cost In-House Re-formulated Brain Heart Infusion Medium for Effective Planktonic Growth and Early Detection of Bloodstream Bacterial Pathogens

Jonathan Hira[1], Nasib Bin Mahbub[1], Jawad Ali[1], Rafi Ahmad[1,2*]

[1] Department of Biotechnology, University of Inland Norway, Holsetgata 22, 2317, Hamar, Norway

[2] Institute of Clinical Medicine, Faculty of Health Sciences, UiT - The Arctic University of Norway, Hansine Hansens veg 18, 9019, Tromsø, Norway

* Correspondence: rafi.ahmad@inn.no

Supplementary Table 1. List of species-specific primers used in this study

**Target Species**	**Target**	**Product size (bp)**	**Species specific primer**	**Refe rence**
E. coli NCTC 13441	Universal stress protein- *uspA*	~ 884	F- 5' CCGATACGCTGCCAATCAGT 3'<br>R- 5' ACGCAGACCGTAGGCCAGAT 3'	(Ana stasi et al., 2010)
S. aureus CCUG 17621	Thermonucl ease *-nuc*	~ 65	F- 5' GGGTTGATACGCCAGAAACG 3'<br>R- 5' TGATGCTTCTTTGCCAAATGG 3'	(Ahm adi et al., 2023)
K. pneumoniae CCUG 225T	Hemolysin *khe*	~ 486	F- 5' TGATTGCATTCGCCACTGG 3'<br>R- 5' GGTCAACCCAACGATCCTG 3'	(Jian-li et al., 2017)
A. baumannii CCUG 19096T	DNA gyrase *gyrA*	~344	F- 5'-AAATCTGCCCGTGTCGTTGGT -3'<br>R- 5'-GCCATACCTACGGCGATACC -3'	(Park et al., 2011)
P. aeruginosa CCUG 17619	Phenazine biosynthesis protein *PhZA2*	~325	F- 5' GTTTACCGACAACCTGGAA 3'<br>R- 5' GCAATAGCCCTGCGGATAC 3'	(Wan g et al., 2022)
E. faecalis CCUG 9997	heat shock proteins groES	~185	F- 5' GGAATTGTTCTTGCATCCGT 3'<br>R- 5' ACAATTAAGTATTCTACGCC 3'	(Teng et al., 2001)

Supplementary Table 2. CFU count comparison between BHI-Blood+ and BD BACTEC™ at different time points for all the strains

Species	Time (Hour)	Culture medium	Replicate 1 (CFU/ml)	Replicate 2 (CFU/ml)
***E. coli NCTC* 13441**	2	BHI-Blood+	1.40×10^5	1.20×10^5
		BD BACTEC™	1.40×10^5	9.00×10^4
	4	BHI-Blood+	1.30×10^6	9.00×10^5
		BD BACTEC™	1.10×10^6	7.00×10^5
***K. pneumoniae* CCUG 225T**	2	BHI-Blood+	2.40×10^5	2.00×10^5
		BD BACTEC™	2.50×10^5	2.00×10^5
	4	BHI-Blood+	3.00×10^6	2.20×10^6
		BD BACTEC™	2.60×10^6	2.70×10^6
***A. baumannii* CCUG 19096T**	2	BHI-Blood+	3.00×10^3	3.00×10^3
		BD BACTEC™	5.00×10^3	5.00×10^3
	4	BHI-Blood+	4.00×10^4	1.00×10^5
		BD BACTEC™	1.20×10^5	6.00×10^4
***P. aeruginosa* CCUG 17619**	2	BHI-Blood+	5.00×10^2	9.00×10^2
		BD BACTEC™	6.00×10^2	5.00×10^2
	4	BHI-Blood+	8.00×10^3	7.00×10^3
		BD BACTEC™	9.00×10^3	1.30×10^4
	6	BHI-Blood+	5.00×10^4	4.00×10^4
		BD BACTEC™	4.00×10^4	3.00×10^4
***E. faecalis* CCUG 9997**	2	BHI-Blood+	1.10×10^5	1.60×10^5
		BD BACTEC™	1.20×10^5	1.40×10^5
	4	BHI-Blood+	5.00×10^5	6.00×10^5
		BD BACTEC™	8.00×10^5	7.00×10^5
***S. aureus* CCUG 17621**	2	BHI-Blood+	2.00×10^2	4.00×10^2
		BD BACTEC™	3.00×10^2	3.00×10^2
	4	BHI-Blood+	1.10×10^4	1.20×10^4

		BD BACTEC™	1.20×10^4	1.00×10^4
	6	BHI-Blood+	5.00×10^5	7.00×10^5
		BD BACTEC™	3.00×10^5	4.00×10^5

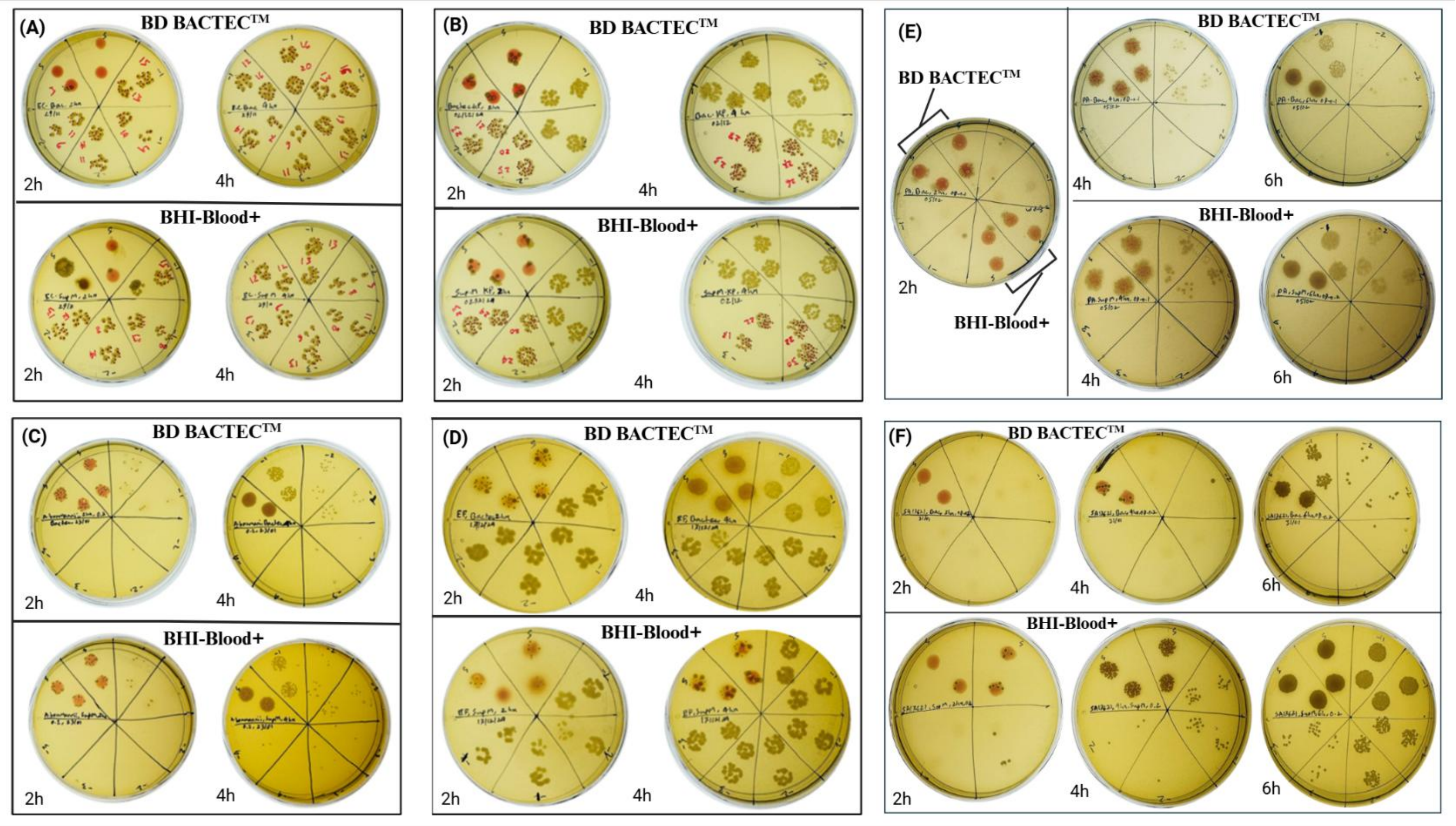

Supplementary Figure 1: Overview of comparative static growth analysis of target species in BHI-Blood+ Media and BD BACTEC™. Colony forming units for individual species are examined on BHI agar plates at different time points (2,4 or 6) after being cultivated at 37°C. (A) *E coli* NCTC13441, (B) *K. pneumoniae* CCUG225T, (C) *A. baumannii* CCUG 19096T, (D) *E. faecalis* CCUG9997 (E) *P. aeruginosa* CCUG17619, (F*) S. aureus* CCUG17621.

Supplementary Table 3. qPCR CT values

Species	Timepoint (hours)	R1	R2	R3	Mean Ct	Std. Dev.
E coli NCTC13441	Isolates	13.07	13.09	13.1	13.09	0.02
	4	22.84	22.74	22.79	22.79	0.05
	24	13.3	13.3	13.32	13.31	0.01
P. aeruginosa CCUG17619	Isolates	14.6	14.6	14.6	14.60	0.00
	4	20.71	20.62	20.65	20.66	0.05
	24	14.9	15	15.06	14.99	0.08
K. pneumoniae CCUG225T	Isolates	30.9	30.3	NA	30.60	0.42
	4	37.44	39.54	NA	38.49	1.48
	24	25.8	26	25.85	25.88	0.10
A. baumannii CCUG 19096T	Isolates	13.63	13.62	13.64	13.63	0.01
	4	17.95	18.07	18.07	18.03	0.07
	24	13.2	13.2	13.26	13.22	0.03
S. aureus CCUG17621	Isolates	14	14	14	14.00	0.00
	4	15.54	15.52	15.49	15.52	0.03
	24	14.4	14.4	14.49	14.43	0.05
E. faecalis CCUG9997	Isolates	13.8	13.8	13.8	13.80	0.00
	4	34.4	32.96	32.24	33.20	1.10
	24	13.6	13.7	13.65	13.65	0.05

Supplementary Table 4. qPCR ΔCT values between 4 hours and 24 hours

Species	Mean_Ct_4	Mean_Ct_24	SD_4	SD_24	ΔCt
A. baumannii CCUG 19096T	18.03	13.22	0.06928203	0.03464102	4.81
E. faecalis CCUG9997	33.2	13.65	1.09981817	0.05	19.55
E.coli NCTC13441	22.79	13.3066667	0.05	0.01154701	9.48
K. pneumoniae CCUG225T	38.49	25.8833333	1.48492424	0.1040833	12.61
P. aeruginosa CCUG17619	20.66	14.9866667	0.04582576	0.08082904	5.67
S. aureus CCUG17621	15.5166667	14.43	0.02516611	0.05196152	1.09

Supplementary Table 5. qPCR ΔCT values between pure isolates and 4 hours

Species	Mean_Ct_Isolates	Mean_Ct_4	SD_Isolates	SD_4	ΔCt
A. baumannii (CCUG 19096T)	13.63	18.03	0.01	0.06928203	4.4
E. faecalis (CCUG9997)	13.8	33.2	0	1.09981817	19.4
E.coli (NCTC13441)	13.09	22.79	0.01527525	0.05	9.7
K. pneumoniae (CCUG225T)	30.6	38.49	0.42426407	1.48492424	7.89
P. aeruginosa (CCUG17619)	14.6	20.66	0	0.04582576	6.06
S. aureus (CCUG17621)	14	15.5166667	0	0.02516611	1.52

Supplementary Table 6. qPCR ΔCT values between pure isolates and 24 hours

Species	Mean_Ct_Isolates	Mean_Ct_24	SD_0	SD_24	ΔCt
A. baumannii (CCUG 19096T)	13.63	13.22	0.01	0.03464102	0.41
E. faecalis (CCUG9997)	13.8	13.65	0	0.05	0.15
Ecoli (NCTC13441)	13.0866667	13.3066667	0.01527525	0.01154701	0.22
K. pneumoniae (CCUG225T)	30.6	25.8833333	0.42426407	0.1040833	4.72
P. aeruginosa (CCUG17619)	14.6	14.9866667	0	0.08082904	0.39
S. aureus (CCUG17621)	14	14.43	0	0.05196152	0.43

Supplementary Table 7. Data retrieved from Oxford nanopore for evaluation of target species detection

Species	Total reads	Mean Read length	No. of reads aligned	Coverage	*E value*
E. coli (NCTC13441)	160057	1745.7	7	0.33	+
P. aeruginosa (CCUG17619)	4210	1999.7	9	0.2	+
K. pneumoniae (CCUG225T)	59953	1708.5	425	13	+
A. baumannii (CCUG 19096T)	49318	1869.3	49	2	+
S. aureus (CCUG17621)	45358	1916.2	4677	95	+
E. faecalis (CCUG9997)	15298	1266.3	NA*	NA*	NA*

NA, not available; +, highly significant

Supplementary References

Ahmadi, A., Khezri, A., Nørstebø, H., and Ahmad, R. (2023). A culture-, amplification-independent, and rapid method for identification of pathogens and antibiotic resistance profile in bovine mastitis milk. *Front. Microbiol.* 13, 1104701. doi: 10.3389/fmicb.2022.1104701

Anastasi, E. M., Matthews, B., Gundogdu, A., Vollmerhausen, T. L., Ramos, N. L., Stratton, H., et al. (2010). Prevalence and Persistence of *Escherichia coli* Strains with Uropathogenic Virulence Characteristics in Sewage Treatment Plants. *Appl. Environ. Microbiol.* 76, 5882–5886. doi: 10.1128/AEM.00141-10

Jian-li, W., Yuan-yuan, S., Shou-yu, G., Fei-fei, D., Jia-yu, Y., Xue-hua, W., et al. (2017). Serotype and virulence genes of Klebsiella pneumoniae isolated from mink and its pathogenesis in mice and mink. *Sci. Rep.* 7, 17291. doi: 10.1038/s41598-017-17681-8

Park, S., Lee, K. M., Yoo, Y. S., Yoo, J. S., Yoo, J. I., Kim, H. S., et al. (2011). Alterations of gyrA, gyrB, and parC and Activity of Efflux Pump in Fluoroquinolone-resistant Acinetobacter baumannii. *Osong Public Health Res. Perspect.* 2, 164–170. doi: 10.1016/j.phrp.2011.11.040

Teng, L.-J., Hsueh, P.-R., Wang, Y.-H., Lin, H.-M., Luh, K.-T., and Ho, S.-W. (2001). Determination of *Enterococcus faecalis groESL* Full-Length Sequence and Application for Species Identification. *J. Clin. Microbiol.* 39, 3326–3331. doi: 10.1128/JCM.39.9.3326-3331.2001

Wang, C., Ye, Q., Jiang, A., Zhang, J., Shang, Y., Li, F., et al. (2022). Pseudomonas aeruginosa Detection Using Conventional PCR and Quantitative Real-Time PCR Based on Species-Specific Novel Gene Targets Identified by Pangenome Analysis. *Front. Microbiol.* 13, 820431. doi: 10.3389/fmicb.2022.820431
